# Stiffness Enhancement in Nacre-Inspired Nanocomposites due to Nanoconfinement

**DOI:** 10.1038/srep16452

**Published:** 2015-11-20

**Authors:** Chen Shao, Sinan Keten

**Affiliations:** 1 Department of Mechanical Engineering, Northwestern University, 2145 Sheridan Road, Evanston, Illinois 60208-3109, United States; 2 Department of Civil and Environmental Engineering, Northwestern University, 2145 Sheridan Road, Evanston, Illinois 60208-3109, United States

## Abstract

Layered assemblies of polymers and graphene derivatives employ nacre’s tested strategy of intercalating soft organic layers with hard crystalline domains. These layered systems commonly display elastic properties that exceed simple mixture rule predictions, but the molecular origins of this phenomenon are not well understood. Here we address this issue by quantifying the elastic behavior of nanoconfined polymer layers on a model layered graphene-polymer nanocomposite. Using a novel, validated coarse-grained molecular dynamics simulation approach, here we clearly show that the elastic properties of layered nanocomposites cannot be described by volume fraction considerations alone and depend strongly on both interfacial energy and nanostructure. We quantify the relative importance of polymer nanoconfinement and interfacial energy on polymer structure and elasticity, and illustrate the validity of our model for two polymers with different intrinsic elastic properties. Our theoretical model culminates in phase diagrams that accurately predict the elastic response of nacre-inspired nanocomposites by accounting for all material design parameters. Our findings provide widely applicable prescriptive guidelines for utilizing nanoconfinement to improve the mechanical properties of layer-by-layer nanocomposites. Our findings also serve to explain why the elastic properties of organic layers in nacre exhibit multifold differences from the native and extracted states.

Nacre, or mother of pearl, is the inner layer of many mollusk shells. It has a brick-mortar type nanostructure consisting of brittle inorganic aragonite platelets and soft organic biopolymer layers. The organic layers are less than 5% by weight, yet they increase toughness by orders of magnitude through a variety of proposed mechanisms such as load transfer through shear deformation, trapping of cracks upon reaching the soft matrix, or toughness amplification by allowing for large deformation and viscoelasticity^1^,^2^. Following on the cue that nacre possesses outstanding properties arising from the layer-by-layer nanostructure, artificial nacre-like nanocomposites have recently been fabricated using a variety of techniques^3^. The elastic modulus and ultimate tensile strength of polymer-clay nanocomposites obtained by layer-by-layer (LbL) assembly^4^ have even exceeded those observed in nacre^5^,^6^,^7^. Advances in synthesis now allow precise nanostructures with polymer layers as thin as 2nm. Ultrathin layers facilitate very high stiffness and toughness by minimizing polymer volume fraction while retaining the beneficial characteristics^8^.

The confinement of the polymer layers to ultra-thin dimensions makes it challenging to measure the properties of the soft phase. Linear elastic fracture mechanics considerations suggest that the modulus mismatch between the soft and hard phases must be high to contribute to several toughening mechanisms proposed for nacre^9^,^10^,^11^,^12^,^13^,^14^,^15^. Assuming bulk properties for the soft phase can be misleading because the *interfaces* (boundary surface formed between two different phases in a material) with the hard layers give rise to soft phase domains that diverge from the bulk behavior, which is attributed to *interphase* (transition region between two different phases in a material) formation in nanocomposites. Systematic studies on synthetic nanocomposites reveal that several molecular mechanisms, such as topological constraints induced by impermeable platelets, chain adsorption onto surfaces, and dispersion of nanoinclusions influence the mechanical properties of polymer nanolayers^16^,^17^,^18^. These mechanisms are collectively called nanoconfinement effects, and it hypothesized that they may contribute to the exceptionally high elastic response observed in nacre and nacre-inspired systems. Most of the circumstantial evidence for these effects comes from polymer thin films, which exhibit drastic changes in glass transition behavior due to substrate effects, in analogy with layered nanocomposites^19^,^20^,^21^,^22^,^23^. Nanoconfinement of polymer thin films near hard surfaces with strong adhesion energy gives rise to a higher apparent glass-transition temperature (*T*_*g*_), and elastic properties may change both above and below (*T*_*g*_)^24^,^25^,^26^,^27^. The length-scale over which these properties change, the so-called interphase width, is a key factor governing the viscoelastic properties of nanocomposites, although it is difficult to measure it experimentally^15^,^24^,^28^,^29^,^30^,^31^,^32^. Such interphases also exist in nacre, as evident from nanoindentation experiments, AFM imaging, and finite element modeling^12^,^33^,^34^,^35^,^36^,^37^. These investigations concur on the observation that the elastic modulus of organic layers are higher than what is anticipated for the organic layers, lying broadly in the range 2 – 40 GPa^12^,^33^,^34^,^35^,^36^,^37^. Conversely, studies on the actual bulk properties of the organic layers reported an elastic modulus of 100 Pa^38^ to 20–100 MPa^39^ for the organic phase using different experimental techniques. We note that the micromechanics models and measurements employed for these analyses often do not account for anisotropy that is likely to occur in such systems, as observed in semicrystalline polymer-clay nanocomposites^40^. Thus, these values obtained are considered to be representative isotropic equivalent material constants. While it is clear that the organic layers confined in their nanolayers in nacre and nacre-inspired nanocomposites exhibit significant differences under nanoconfinement, how these properties depend on factors such as layer thickness and interfacial energy remains to be established.

In this article, we aim to study the nanoconfinement effect using a novel coarse-grained molecular dynamics (CGMD) model of nacre-inspired poly(methyl methacrylate) (PMMA)/graphitic systems, as recently synthesized and studied in experiments^41^. For hydrated organic layer in nacre, the elastic modulus is reported to be close to PMMA’s elastic modulus. Thus, the PMMA/multi-layer graphene system has similar constitutive behavior as the nacre constituents. The simulation approach utilizes recent advances in mesoscale modeling of materials, namely the development of coarse-scale models that can capture the mechanical properties of multi-layer graphene^42^ and methacrylate polymers^43^,^44^ at length and time scales inaccessible to all-atom MD simulations. This ability allows us to efficiently carry out size-dependence studies using models validated by experiments. Here, we first discuss the details of the modeling approach. We follow up with results focusing on the properties of the soft phase, interrogating size-dependence along with interfacial energy. Finally, we summarize our conclusions and present analytical models that provide guidelines for designing and optimizing material properties in nacre-inspired systems.

## Methods

### Coarse-Grained Models

#### 2-bead per monomer model for PMMA

The coarse-grained potential for PMMA used in this study is based on a generalized CG force field that we developed in a recent study^43^. The bonded parameters were derived from all-atomistic probability distributions of local structural metrics, and long-range interactions were based on molecular mobility and density measurements as described in the original work^43^. Each monomer in PMMA is modeled as 2 bead groups in our CG model: the backbone methacrylate group “A” (C_4_O_2_H_5_) and side-chain group “B”. The bond stretching, angle bending, and dihedral interactions in the CG model are developed by matching them to respective atomistic probability distribution functions using the inverse Boltzmann method. We employ a Gromacs-style 12−6 Lennard-Jones (LJ) potential to model the cohesive nonbonded interactions between beads excluding the nearest bonded neighbors:


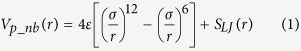


where *ε* is the depth of potential well and *σ* is the point at which the potential crosses the zero energy line. S_LJ_(r) is a polynomial function that provides the interaction a smooth transition to zero from *r*_*inner*_ = 12 Å to *r*_*outer*_ = 15 Å. The parameters have been calibrated to match the experimental density at room temperature and the glass transition temperature, *T*_*g*_, of bulk PMMA, resulting *ε*_*AA*_ = 0.5 kcal/mol, *σ*_*AA*_ = 5.5 Å for backbone beads and *ε*_*BB*_ = 1.5 kcal/mol, *σ*_*BB*_ = 4.42 Å for sidechain beads, which yeild density of 1.15 g/cm^3^ and *T*_*g*_ of 385 K for bulk PMMA. Additionally, the model is validated using experimental data on the Flory−Fox constants for PMMA that define the molecular weight dependence of *T*_*g*_, which our model readily captures with no additional empirical input.

#### Coarse-grained Model for Multi-layer Graphene

The details of the graphene model are explained in our earlier work^42^ and are briefly summarized here. We cluster 4 atoms into one bead and the potential energy can be written as:


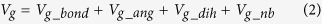


where *V*_*g_bond*_, *V*_*g_ang*_, *V*_*g_dih*_, *V*_*g_nb*_ represent the total bond, angle, dihedral, and pair wise non-bonded interactions. The functional form of the interactions are as follows:


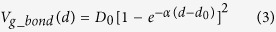



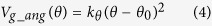



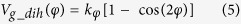



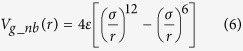


where *D*_*0*_ is the depth of the bond potential well, *α* is related to the width of the potential well of the bond, *d*_*0*_, *θ*_*0*_ are equilibrium bond and angle. *k*_*θ*_, *k*_*φ*_ are spring constants of angle and dihedral interactions. *ε* is the depth of the non-bonded potential well and *σ* determines the equilibrium distance between two non-bonded beads (*r*_*eq*_ *=* *2*^*1/6*^*σ*). Based on the geometry of the mapping, in our system: *d*_*0*_ = 2.8 Å, *θ*_*0*_ = 120°, and *σ* = 3.46 Å. The rest of the parameters are calibrated based on material properties as: *D*_*0*_ = 196.38 kcal/mol, *α* = 1.55 Å, *k*_*θ*_ = 409.40 kcal/mol, *k*_*φ*_ = 4.15 kcal/mol, *ε* = 0.82 kcal/mol. For the non-bonded interactions, the cut off distance is calibrated as 12 Å and the depth of the energy is calibrated such that the interlayer adhesion energy is 260 mJ/m^2^. As discussed in the original work, all of these values are in close agreement with data from experiments and density functional theory calculations^42^. This graphene CG model yields, for a monolayer, an elastic modulus of 900 GPa, failure strength of 81 GPa, and in-plane shear modulus of ~2 GPa in zigzag and ~1.5 GPa in armchair pulling directions, all in good agreement with experimental results^42^. The interlayer shear modulus is about 2 GPa for the system studied here, but depending on the stacking configuration, it can be much lower. A key feature of the model is its ability to predict the elastic and plastic response of multi-layer graphene, and features such as superlubricity, where a drastic reduction in shear resistance can be observed at specific stacking arrangements. Our CG model can quantitatively capture complex mechanical behavior such as non-linear elasticity, buckling of the sheets under large shear deformation or anisotropy in the zigzag and armchair directions for large-deformation and fracture, and is thus well equipped to model graphene properties in nanocomposite materials.

### Methodology for the Coarse-Grained Molecular Dynamics Simulations

**Figure 1:**
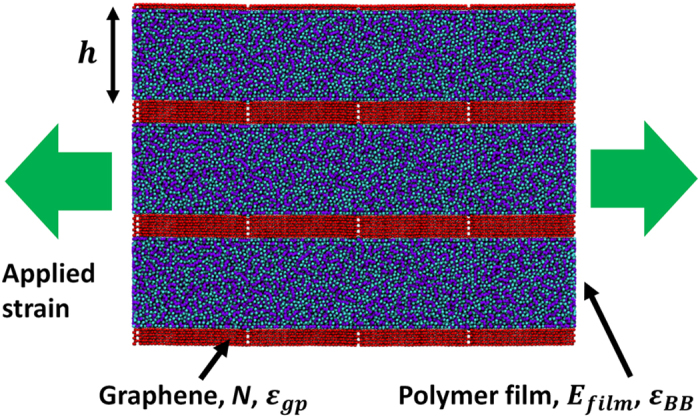
Schematics of the system, periodic boundary conditions are employed in all 3 axes. *h* is the thickness of polymer layer. *N* is the number of graphene layers. *E*_*film*_ is the elastic modulus of polymer layer. *ε*_*BB*_ is the sidechain cohesive interaction parameter. *ε*_*gp*_ is the interfacial interaction parameter.

We use the Large-scale Atomic/Molecular Massively Parallel Simulator (LAMMPS), widely used open sourced simulation package to carry out our CGMD simulations. The systems are composed by 2 phases: the relatively soft polymer phase and the hard graphene phase. Periodic boundary conditions (PBC) are applied in all 3 directions (x,y,z), so that with the two phases stacked together, the system constitutes infinitely long layers in both the x and y directions, and an infinite repeating bilayer structure of alternating graphene and polymer phases in the z direction. Thereby, a simplified, uniform layer-by-layer structure inspired from the nanostructure of nacre can be formed. The graphene phase has N finite graphene sheets that are 9.7 nm long (x) and 50.96 nm wide (y), containing 2 graphene flakes of equal size in each sheet plane. With PBC, the resulting lateral spacing between the flakes in the y direction is ~4.8 Å. When N > 1, the sheets, AB stacked in a staggered fashion, are shifted by one half of the length of the graphene flake with respect to the neighboring (above or below) layers, resulting in an overlapping percentage of 50%. In the polymer phase, PMMA chains with 100 monomers per chain are first equilibrated at 800 K and then slowly cooled down to room temperature. The polymer films with different thickness *h* are then placed onto the graphene phase to create the layered systems. The interaction between the graphene and polymer is captured by the LJ 12-6 potential:


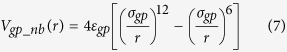


where *ε*_*gp*_ is the depth of the Lennard-Jones potential well for graphene-polymer interaction strength and *σ*_*gp*_ is the point where potential crosses zero point line. The cutoff distance is set to be 15 Å. Previous studies have shown that stronger interfacial interactions lead to higher T_g_ and elastic modulus near interface seen in supported thin films and nanocomposites^44^,^45^,^46^,^47^. For graphene derived materials, this can be straightforwardly achieved through surface functionalization, as in graphene oxide. To better understand the impact of interfacial interaction strength on the elastic response, *ε*_*gp*_ = 0.5, 1.25, 2.0 kcal/mol are used in our modeled nanostructures so that the interfacial energies represent different types of interfaces from weakly bonded graphene polymer interface to highly adhesive graphene oxide polymer interface where no-slip condition at interface is ensured. The interfacial energy between graphene and soft layers scales linearly with *ε*_*gp*_ and is 0.08 J/m^2^, 0.25 J/m^2^ and 0.45 J/m^2^ respectively. For weak interfacial interaction strength, the calculated adhesion energy is comparable to experimentally measured graphene polymer adhesion energy^48^. Our interfacial energy systems ensure no-slip boundary conditions at polymer graphene interface, which can be achieved experimentally with surface functionalization as in the case of graphene oxide. To construct our modeled structures, we select *N* = 1, 2, 5, 8, corresponding to graphene phase thickness of 0.34nm, 0.68nm, 1.7nm, and 2.72nm. We choose *h* = 2 nm, 5 nm, 10 nm, 20 nm, 40 nm for polymer phase. We also wish to elucidate whether the confinement effect depends on the type of the polymer used, specifically its bulk elastic properties. Therefore we reduce the cohesive interaction in sidechain groups by adjusting the *ε*_*BB*_ parameter in our CG PMMA model to 0.1 kcal/mol. This results in a hypothetical polymer that has a lower glass transition temperature and a lower modulus for the polymer phase^44^,^45^. In total, 60 systems with varying *h*, *N* and interfacial interaction *ε*_*gp*_ are studied for both low cohesive energy and high cohesive energy polymer nanocomposites. A schematic of system is shown in Fig. 1.

The densities of the modeled systems are calculated to be 1.19 ± 0.03 g/cm^3^, which is the same as the density in bulk polymer phase. It is therefore considered that the confinement on polymer phase does not significantly change the average density of the polymer. For equilibration, we start with a fast push off phase using a soft potential to randomize chain configurations, and then equilibrate the system under 800 K for 6 ns. During the equilibration process, the cut off distance of our CG model is set to be *2*^*1/6*^*σ* so that only repulsive interactions are allowed for the polymer phase to overcome conformational energy barriers and achieve equilibrium at high temperature. The system is then cooled down to 300K and equilibrated for 2 ns with the full potential described in Equations 1, 2, 3, 4, 5, 6, 7. Polymer chains in our modeled nanostructures have 100 monomers per chain and are below the entanglement length measured in experiments^49^, and thus find their relaxed conformations more readily. Here we ensure that the polymer chains have converged conformations by initializing polymer chains with desired end-to-end distances and monitoring the mean-square internal distances (MSID) in polymer chains during the equilibration procedure. Steady states are attained by monitoring the convergence in MSID curves for the polymer layers^50^. After equilibration runs, a strain-controlled uniaxial tensile test is performed by deforming the simulation box in the y direction at a strain rate of 2 × 10^8^ s^−1^. This high strain rate is inherent to MD simulations, which includes dynamical information usually on ps or ns timescales. We note that since the polymers used are below their glass-transition temperature, strain rate effects on the measured moduli are not expected to be very large. We also note that high strain rates are highly relevant to ballistic impact and other protection applications where nacre-inspired systems could potentially be utilized. In such cases, the deformations occur athermally, and strain rate effects are minimal for modulus measurements, which are governed chiefly by the cohesive interactions. Previous molecular dynamics studies show that employing a strain rate between 0.4 and 40 × 10^8^ s^−1^ yields consistent results for elastic modulus calculations in other systems^51^. During the deformation, the pressure is kept at zero in all directions except for the loading direction. Virial stress is computed for each atom in the simulation box and is averaged over all atoms in polymer phase to get stress in confined polymer film. Elastic modulus of polymer phase is then computed from the slope of a linear fit to the stress-strain curve with strain *ε* = 0–0.015.

## Results and Discussion

### Stress strain curves in modeled systems

**Figure 2:**
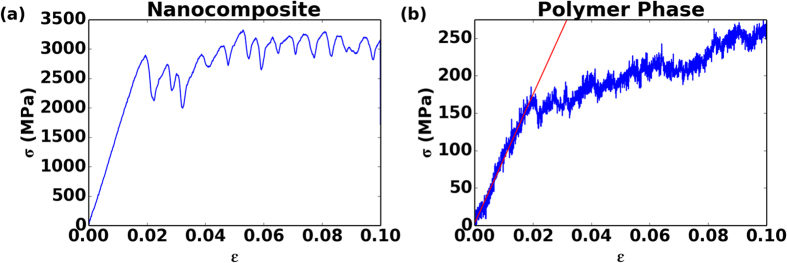
Typical stress strain curves for (**a**) the nanocomposite and (**b**) the polymer phase the tensile deformation of graphene/polymer nanocomposite with *ε*_*gp*_ = 2.0 kcal/mol, *N* = 2, *h* = 20 nm, shown for tensile deformation up to 10%. We use the segment up to 1.5% strain to fit the slope to obtain the elastic modulus E. Multiple peaks in total stress strain curve correspond to stick-slip events of graphene sheets in the hard phase.

First, we present results from the constant strain rate tensile testing simulations, which provide insights into the mechanical response of the system. We focus initially on how the thickness of the polymer layer influences elastic properties. For this purpose, we present results from a series of computational thought experiments where we vary the nanostructure of the multilayer system by controlling both *h*, the thickness of the polymer layer, and *N*, the thickness of the graphene layer as defined by the number of sheets. A typical stress strain curve of the nanocomposite system as well as the polymer phase is shown in Fig. 2(a,b). The overall stress-strain behavior of the nanocomposite indicates that the material can be considered linear elastic up to 1.5% strain. Shortly after the linear elastic region, multilayer graphene starts to yield due to interlayer sliding between graphene sheets and stick-slip events occur between graphene flakes, marking the onset of a plastic regime. This plastic deformation mechanism is associated with a post-yield plateau in the stress-strain curve that exhibits repeated peaks and valleys as the sheets slide. Meanwhile, the interfacial energy between the polymer and graphene phase is large enough such that the chain ends are physisorbed and move with the graphene layers. The large shear stresses that develop in the soft layers eventually give rise to graceful failure of the material. Given the complexity of the mechanisms involved and their size-dependence, here we limit our focus to the small deformation regime, that is, the linear elastic region, where the nanoconfinement effects are already not well understood. Specifically, we aim to investigate the combined influence of geometric nanoconfinement and interfacial energy on the elastic properties of the polymer phase.

### Structural characteristics of the confined polymer layer

**Figure 3:**
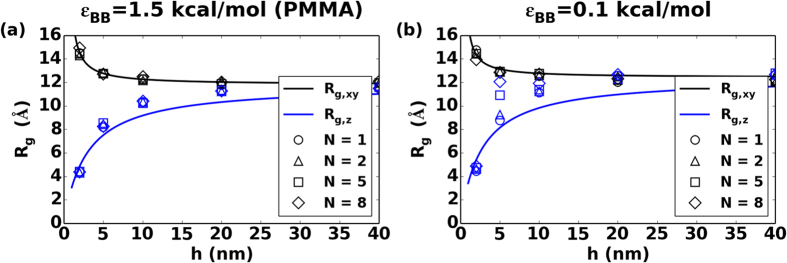
Plot of gyration tensor of confined polymer with *ε*_*BB*_ = (**a**) 1.5 kcal/mol (PMMA) and (**b**) 0.1 kcal/mol in both in-plane *R*_*g,xy*_ and out-of-plane *R*_*g,z*_ directions vs polymer phase thickness *h* for systems with interfacial interaction strength *ε*_*gp*_ = 2.0 kcal/mol. *N* is the number of graphene sheets in graphene phase.

The first key question we ask here is whether the confinement by graphene layers induces structural changes in the polymer layer. If any changes occur in the structural arrangement of the polymer chains, one could potentially correlate these with the changes in the mechanical behavior as well^52^,^53^,^54^,^55^. In order to study the confinement effect on the conformation of polymer chains in the soft phase, we calculate the gyration tensor for each polymer chain, and plot the average value against polymer layer thickness, *h* in Fig. 3. *R*_*g*_ is a measure of the molecule’s size and orientation in specified directions and is defined as:


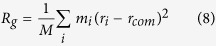


where *M* is the total mass of the system, *m*_*i*_ and *r*_*i*_ are the mass and position of each atom in the system, *r*_*com*_ is the center of mass for all the atoms. In our systems, z-axis is orthogonal to the plane of layers of graphene and polymer. Therefore, two directions are of interest here to study the confinement effect: in-plane (parallel to *xy* plane, or equivalently the plane of the graphene sheets) and out-of-plane (perpendicular to *xy* plane). *R*_*g,xy*_ is the square root of average value of both *x* and *y* direction *R*_*g*_ squared. The confinement effect on structure is clear in our bilayer systems: as *h* reduces, both in-plane and out-of-plane *R*_*g*_ deviates from *R*_*g*_ in bulk polymer: *R*_*g,xy*_ increases and out-of-plane *R*_*g,z*_ decreases. Calculated *R*_*g*_ results suggest that the strength of the interfacial interaction does not drastically change the average structural confirmations for the cases studied here. Instead, the soft layer thickness, *h,* and the associated topological changes seem to be the more dominant factor governing *R*_*g*_, as all 3 studied interfacial energy systems produce very similar results. Therefore, for clarity, only systems with *ε*_*gp*_ = 2.0 kcal/mol are shown in Fig. 3. Each data point in Fig. 3 is an averaged value over 5 distinct simulation runs. The errors are small compared to the value and therefore omitted in Fig. 3 for clarity.

### Elastic modulus for confined polymer phase

**Figure 4:**
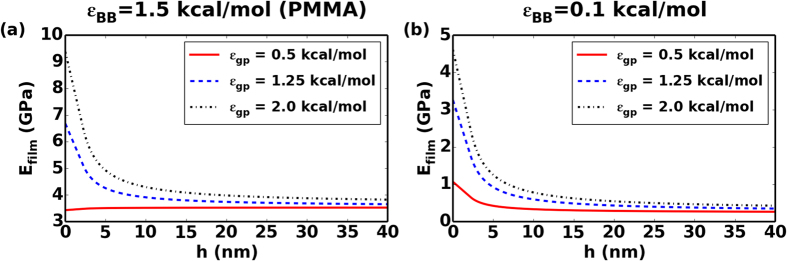
Model predictions of the elastic modulus of the confined polymer phase using Equation 11 for polymer with *ε*_*BB*_ = (**a**) 1.5 kcal/mol (PMMA) and (**b**) 0.1 kcal/mol with interfacial interaction strength *ε*_*gp*_ = 0.5, 1.25, 2.0 kcal/mol. *h* is the thickness of confined polymer phase, *E*_*film*_ is the elastic modulus of confined polymer phase.

The next question that remains to be answered is whether the structural changes observed in the confined soft layers directly correlate with changes in mechanical properties that are associated with nanoconfinement. For this purpose, we compare the elastic moduli of the polymer in the bulk (*E*_*bulk*_) and nanoconfined (*E*_*film*_) phases, and again systematically map out the effects of material nanostructure. To calculate elastic modulus *E*_*bulk*_ for bulk phase of polymers, we perform tensile test on systems with periodic boundary conditions and obtain the slope of a linear fit to the stress-strain curve with strain *ε* = 0–0.015, averaging results from 5 distinct simulation runs yields 3.50 ± 0.21 GPa and 0.30 ± 0.11 GPa for polymer with *ε*_*BB*_ = 1.5 kcal/mol (PMMA) and 0.1 kcal/mol (low cohesive interaction polymer) respectively in our CGMD model. Figure 4 illustrates how the *E*_*film*_ scales with *h* under nanoconfinement for both material systems and degree of polymerization. For clarity, calculated elastic modulus data points are omitted in Fig. 4, only predictions from our analytical model (Eq. 11) are presented. Detailed plots containing both elastic modulus data points and prediction curves from analytical models are available in the Supporting Information. For the weak interfacial interaction cases with *ε*_*gp*_ = 0.5 kcal/mol, the elastic modulus of the PMMA layer does not see an increase from its bulk value but an increase of over 130% for low cohesive polymer layer with *ε*_*BB*_ = 0.1 kcal/mol is still observed. For strong interfacial interactions *ε*_*gp*_ of 1.25 kcal/mol and 2.0 kcal/mol, as the thickness of soft layer *h* decreases from 40 nm to 2nm, the elastic moduli of confined PMMA layer increases by 50% and 90% respectively. The trend is similar in polymer layers with *ε*_*BB*_ = 0.1 kcal/mol, but in this case the elastic modulus increase is much more significant, ranging from roughly 5 to 8 times the bulk values. The most interesting observation arising from this comparative analysis is that although the elastic modulus of the polymers studied here differs 10 times in the bulk phase, the values are much closer in the confined state, which shows the importance of the confinement effect for soft polymers. Our simulation results compare well with recent experimental studies on elastic modulus of supported PMMA thin films using nanoindentation techniques indicating increment in elastic modulus with decreasing film thicknesses^56^. Thus, at a very high degree of confinement, common in many synthetic nacre-inspired systems, the degree of confinement rather than polymer chemistry may be the most important factor governing the in-plane elastic response.

**Figure 5:**
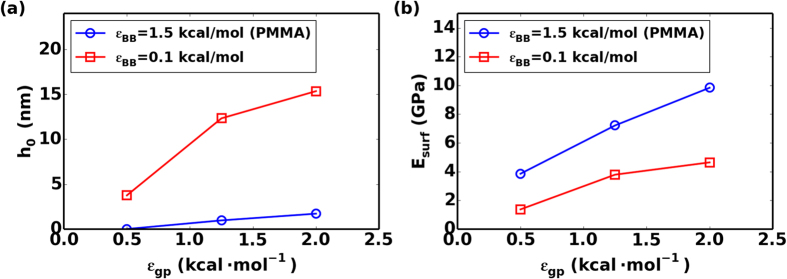
Plot of estimated (**a**) *h*_*0*_ and (**b**) *E*_*surf*_ vs interfacial interaction strength parameter *ε*_*gp*_.

To quantitatively describe the effect of confinement on the elastic moduli of polymer phase, we propose the following model to capture the relationship between elastic moduli and film thickness:


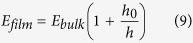


where *E*_*film*_ is the effective elastic modulus of the confined polymer phase, *E*_*bulk*_ is the elastic modulus of bulk phase of polymer, *h* is the thickness of the confined films and *h*_*0*_ is a fitting parameter that determines how rapidly the elastic modulus converges to the bulk value for confined polymer films. For the same type of polymer, the fitting parameter *h*_*0*_ is similar for different values of *N* but depends on interfacial energy as well as the soft layer thickness. In Fig. 5a, we the average *h*_*0*_ across all *N* values for each systems to show the trend with interfacial energy. The growth in *h*_*0*_ with increasing interfacial energy is clear from this analysis. For the high cohesive energy polymer with *ε*_*BB*_ = 1.5 kcal/mol (PMMA), *h*_*0*_ = 0 nm, 0.96 nm, 1.71 nm and for the polymer with *ε*_*BB*_ = 0.1 kcal/mol, *h*_*0*_ = 3.75 nm, 12.33 nm, 15.36 nm for the three values of *ε*_*gp*_ studied respectively. Taking our nanostructures with strong interfacial interaction strength *ε*_*gp*_ = 2.0 kcal/mol as an example, for a confined PMMA film, a 17.1 nm thick film would have an elastic modulus that is within 10% of the bulk value. On the contrary, this thickness increases to 153.6 nm for the low cohesive polymer with *ε*_*BB*_ = 0.1 kcal/mol.

Based on this analysis, one may ask whether the 1/*h* scaling relationship between *E*_*film*_ and thickness *h* identified here has any physical basis. Here we attempt to provide an explanation for this observation using simple composite concepts. On the basis of chain segment order parameter spatial distribution (details in supporting information) in our confined soft layers, we employ a composite bilayer model to quantify the thickness dependence of *E*_*film*_ and justify the best-fit scaling. Based on our finding that the structure properties of confined polymer phase approach bulk-like when one proceeds 2*R*_*g*_ distance away from graphene polymer interface (details in supporting information), here we define an interface region *h*_*int*_ of 2*R*_*g*_ distance from graphene polymer interface. Beyond this region, we assume that the properties converge to bulk like properties and the chains cannot sense the interfaces directly. With this simplification, the confined polymer film with thickness *h* can be considered to be composed of 2 interface layers with thickness *h*_*int*_ and 1 interior layer with bulk like properties. In the interface layer, the elastic modulus can be considered as:


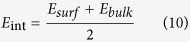


where *E*_*surf*_ is the upper limit of elastic modulus at graphene polymer interface when confinement effect is infinite or *h* → 0. As the distance from graphene polymer interface increases beyond the 2*R*_*g*_ limit, the modulus can be considered as *E*_*bulk*_ for the interior layer. If the film thickness *h* is less than 2*h*_*int*_, this means that the effects of the two interfaces are pervasive throughout the film and there is no interior bulk-like region. Following this picture, the polymer phase modulus *E*_*film*_ can be described as:


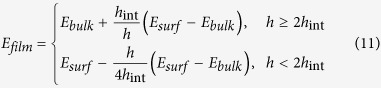


Further investigation in Equation 11 reveals that when *h* > 2*h*_*int*_, this bilayer composite model resumes to our empirical model in Equation 9. By equating Equation 9 and 11, we can define *E*_*surf*_ in terms of *h*_*0*_ using our simulation data simply as:


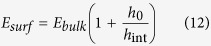


Results from Fig. 5(b) shows that *E*_*surf*_ increases with interfacial interaction strength in confined polymer layers with *ε*_*BB*_ = 1.5 kcal/mol (PMMA) and 0.1 kcal/mol. It should be noted that despite the huge difference in elastic response of bulk PMMA and low cohesive polymer with *ε*_*BB*_ = 0.1 kcal/mol, i.e. 10 times difference in modulus, these two polymer films have comparable surface moduli under nanoconfinement (only ~2 fold difference). This is because the surface moduli presumably depends most strongly on the graphene polymer interaction strength compared to other factors. Comparing Fig. 5(a,b) suggests a similar trend for both *h*_*0*_ and *E*_*surf*_ in variables as expected.

These results ascertain that nanoconfinement greatly alters properties of polymer layers and quantify the size-effects associated with layer-by-layer systems. Our nacre inspired model for elastic modulus of the confined polymer phase successfully captures the CGMD simulation results and provides simple guidelines for designing nacre inspired nanocomposite materials. The key insight here is that the interchain molecular interaction of the confined polymer, which governs the thickness of the interface region, governs *h*_*0*_ in our model and determines how fast the confinement effect changes with changing *h*. Adhesive interaction at the interface is another key factor to consider in such nanocomposites since it influences *E*_*surf*_ in the analytical model and governs the effectiveness of confinement in changing the elastic response of the polymer phase.

**Figure 6:**
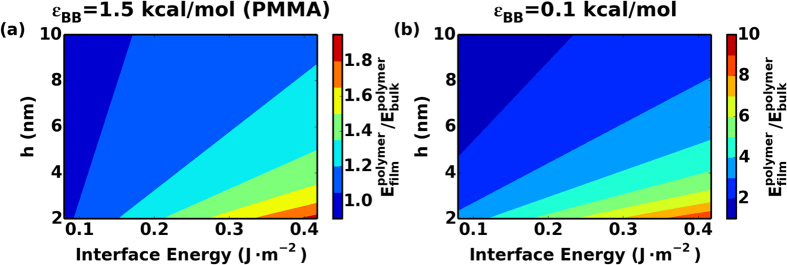
Model predicted *E*_*film*_ using Equation 11 normalized by elastic modulus of polymer with *ε*_*BB*_ = (**a**) 1.5 kcal/mol (PMMA) and (**b**) 0.1 kcal/mol, *h* is the thickness of confined polymer phase.

The effect of both confinement and interfacial energy on elastic moduli of the polymer phase can be best reflected in a phase diagram of *E*_*film*_ in Fig. 6, where we utilize our proposed model in Equation 11 to predict *E*_*film*_ with different confinement thickness *h* as well as interfacial interaction strengths. For each film thickness *h*, we first calculate *E*_*film*_ in systems with *ε*_*gp*_ = 0.5, 1.25, 2.0 kcal/mol using Equation 11 and then linearly extrapolate *E*_*surf*_ values for other values of *ε*_*gp*_. In Fig. 6, the predicted *E*_*film*_ is normalized with the elastic modulus of the bulk polymer. This analysis illustrates that the confinement effect is much stronger in low cohesive energy polymer layers as even with low interfacial energies, nanoconfinement still results in a significant increase in the elastic moduli of polymer phase. It should be noted that in experiments, it is extremely difficult to increase interfacial energy without compromising other properties of the materials. For example, in our PMMA/graphitic systems, functionalizing graphene sheets, as in the case of graphene oxide, can increase the interfacial energy. However, the modulus of graphene oxide decreases monotonically with degree of functionalization due to the breaking of perfect sp^2^ carbon network^57^,^58^. The elastic modulus gain from the confined soft layers is on the order of few GPas and may not be enough to overcome modulus loss of tens and hundreds of GPas when increasing the degree of functionalization in graphene polymer nanocomposites. Therefore, from materials by design point of view, maintaining a high modulus in the hard layer while achieving large interfacial interactions seems to be crucial. Overall, the trends predicted here with our simulations agree very well with a very recent experimental study on graphene oxide PMMA nanocomposites, where the composite modulus nonlinearly overshoots the rule of mixtures predictions when the polymer layer thickness is reduced to tens of nm^59^.

**Figure 7:**
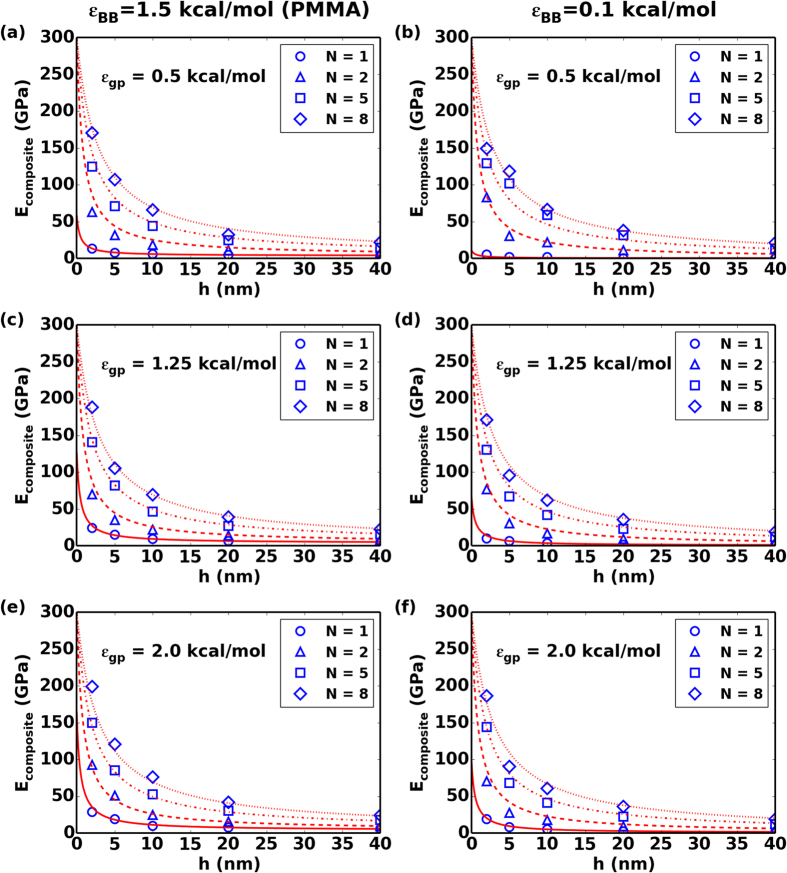
CGMD calculated and composite rule of mixture model (with*E*_*g*_ = 300GPa, *E*_*film*_ predicted using Eq. 11) elastic modulus of nanocomposite for polymer with *ε*_*BB*_ = 1.5 kcal/mol (PMMA) (panel a, c, e) and 0.1 kcal/mol (panel b, d, f) with interfacial interaction strength *ε*_*gp*_ = 0.5, 1.25, 2.0 kcal/mol. *N* is the number of graphene sheets in graphene phase, *h* is the thickness of confined polymer phase, *E*_*composite*_ is the overall elastic modulus.

The modulus of multilayer graphene phase calculated from each system shows that changing number of graphene sheets N does not change the overall elastic modulus of graphene phase when N>1. The modulus is calculated to be *E*_*g*_ ~ 300 GPa, which is in agreement with simulation results on multilayer graphene sheets from our previous study^42^. For systems with N = 1, the calculated elastic modulus of graphene phase is lower due to sheet discontinuities, and depends on the graphene polymer interfacial interaction strength (details in supporting information). Regardless, the elastic modulus can be estimated by using a rule of mixtures using our predictions for nanoconfinement and interfacial energy effects. Figure 7 summarizes the elastic modulus predictions for the whole nanocomposite using our simple model. In this particular system, the much stiffer graphene phase dominates the overall elastic response of the nanocomposite. In many biological and bio-inspired nanocomposites, the hard phase materials possess much a lower elastic response than graphene and interfacial energy can be very high through the use of strong electrostatic interactions. Additionally, our analysis on lower cohesive forces between polymers also serves to emulate hydrated systems where a lower bulk modulus but a greater increase in the confined modulus is likewise anticipated. Thus, the nanoconfinement effects seen here are likely conservative estimates and a much greater contribution from the stiffening of the soft polymer phase can be anticipated in certain relevant cases.

## Conclusions

In this work, we utilized coarse-grained molecular dynamics simulations to systematically study the nano confinement effect on the elastic modulus of the confined polymer phase in nacre inspired nanocomposite materials. Structural characterization of the confined polymer phase illustrated that the graphene phase leads to highly aligned polymer chains near the graphene/polymer interface region. Elastic modulus calculation shows that a high degree of confinement increases the elastic modulus of the polymer phase by as much as 2–6 times, depending on the type of the polymer. These results provide fundamental into how the elastic response of the polymer is altered tremendously under confinement compared to the unconfined state, especially at length scales below 5 nm, which is becoming relevant with more recent synthesis approaches to nacre-inspired systems. Our analytical model physically explains the effect of confinement arising from the hard-soft materials interface and quantitatively captures the effect of confinement on the modulus of the polymer layer. In the context of materials by design, our work serves as a guideline to fabricate nacre-mimetic nanocomposites with optimized elastic properties. Utilizing the same methods used in this article, fracture toughness of these nanocomposites could further be studied and provide a complete overview of materials by design approach. The CGMD approach laid out in this study could also be extended to analyze the mechanical behavior of other 2D materials in nanocomposites, and should be straightforward to generalize to other materials systems inspired from nacre.

## Additional Information

**How to cite this article**: Shao, C. and Keten, S. Stiffness Enhancement in Nacre-Inspired Nanocomposites due to Nanoconfinement. *Sci. Rep.* **5**, 16452; doi: 10.1038/srep16452 (2015).

## Supplementary Material

Supplementary Information
