# Supplementary material for: Stiffness Enhancement in Nacre-Inspired Nanocomposites due to Nanoconfinement
Source: Sci Rep. 2015 Nov 20;5:16452. doi: 10.1038/srep16452 (PMC4653650; doi:10.1038/srep16452)
Supplement: Supplementary Information [file srep16452-s1.pdf]

Supporting Information

Stiffness Enhancement in Nacre-Inspired Nanocomposites due to Nanoconfinement

*Chen Shao and Sinan Keten**

Structure analysis on confined soft layers

To quantify the effect of confinement on polymer chain conformation, we calculated Herman's order parameter S for each of the polymer chains and plotted the average value in the bilayer systems in **Figure S1**. The order parameter is defined as:

$$S = \frac{3\langle \cos^2\theta \rangle - 1}{2} \tag{1}$$

where θ is the angle between polymer chain segments and z axis, and ranges from 0 to π. This is done by first computing the dot product of the unit vector joining the centers of the j and $j - 1$ beads of the chain with the vector defining the z axis to get $cos\theta$. $cos^2\theta$ is then averaged over all chain segments to obtain order parameter S for the molecule. If all polymer chains align with the x-y plane, $\theta = \pi/2$, then S = -0.5. On the other hand, if all polymer chains align vertically out of plane, $\theta = 0$, S =1. If the chains are completely randomly oriented, S = 0. With the increasing polymer phase thickness, order parameter increases and approaches 0. This result is in agreement with the R_g results and shows confinement induces in-plane alignment of polymers and a vanishing component of R_g in the orthogonal directional for very thin layers.

Next, we assess whether the changes observed in the structural behavior of the polymer is uniform across the film, or if conformational gradients can be observed as we go away from the interfaces. **Figure S2** presents the spatial distribution of polymer chain segment order parameter in polymer phase. Here, z is defined as the distance of the chain segment center of mass to the graphene/polymer interface. It is evident that in both low and high molecular weight systems, as

one proceeds roughly $2R_g$ (R_g = 11 Å) distance from the interface, the structural properties of the polymer converges to bulk values. These results are consistent with experiment results that R_g of polymer thin film increases as the film thickness reduced below $4R_g$ of bulk phase.[1,2] Previous work on confinement effect on polymers using Monte Carlo modeling also indicated that R_g in a confined polymer thin film will approach bulk like values with $2R_g$ distance away from the interface.[3] Our R_g and order parameter calculation results suggest that structurally, polymer with high and low cohesive interaction, e.g. ε_{BB} = 1.5 kcal/mol and 0.1 kcal/mol, possess similar characteristics. The number of graphene layer N in graphene phase has little impact on the structure of confined polymer phase. Even one layer of graphene is adequate to induce R_g and S change in confined polymer phase, which suggests that this is largely a topological effect where the graphene sheet serves as an impermeable, adhesive wall.

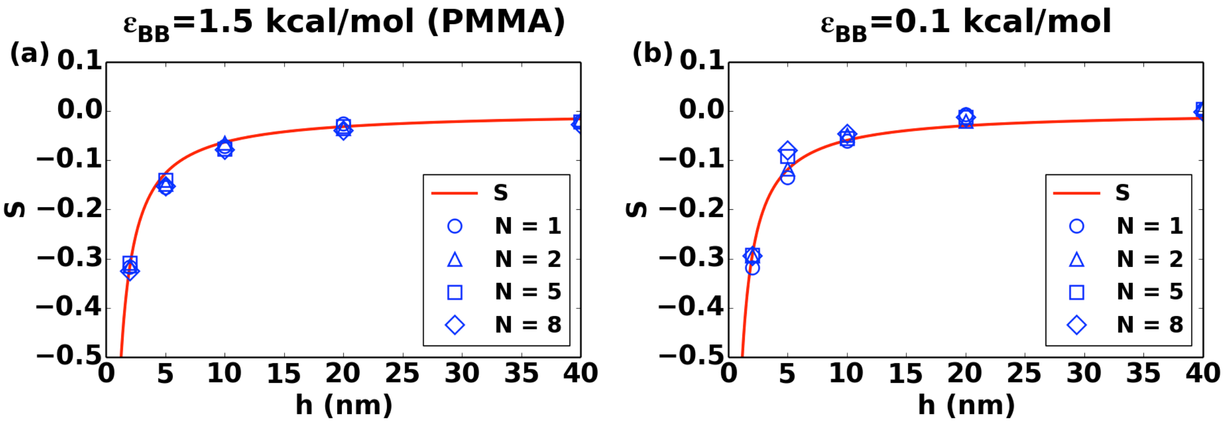

Figure S1. Structural order parameter S of confined polymer with ε_{BB} = (a) 1.5 kcal/mol (PMMA) and (b) 0.1 kcal/mol, plotted against polymer phase thickness h for systems with interfacial interaction strength ε_{gp} = 2.0 kcal/mol. N is the number of graphene sheets in graphene phase.

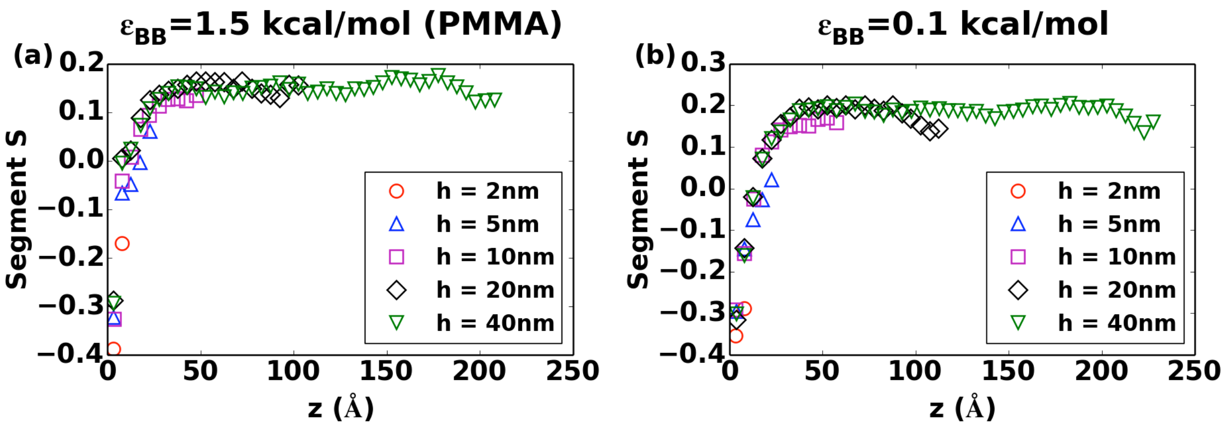

Figure S2. Chain segment order parameter S of confined polymer with ε_{BB} = (a) 1.5 kcal/mol (PMMA) and (b) 0.1 kcal/mol, plotted against segment center of mass distance z to graphene polymer interface for systems with interfacial interaction strength ε_{gp} = 2.0 kcal/mol.

Elastic modulus for confined polymer phase

Figure S3 illustrates how the E_{film} scales with h under nanoconfinement for both material systems and degree of polymerization. Each data point is averaged value over 5 distinct simulation runs. The statistical variation is small for systems with large h, but increases as h reduces. However, the trend for different systems is consistent. For the weak interfacial interaction systems with ε_{gp} = 0.5 kcal/mol, the elastic modulus of the PMMA layer does not see an increase from its bulk value but an increase of over 130% for soft PMMA layer is still observed (Figure S3(*a,b*)). For strong interfacial interactions ε_{gp} of 1.25 kcal/mol and 2.0 kcal/mol (Figure S3(*c,e*)), as the thickness of soft layer h decreases from 40 nm to 2nm, the elastic moduli of confined PMMA layer increases by 50% and 90% respectively. The trend is similar for the polymer with ε_{BB} = 0.1 kcal/mol system (Figure S3(*d,f*)), but in this case the elastic modulus increase is much more significant, ranging from roughly 5 to 8 times the bulk values.

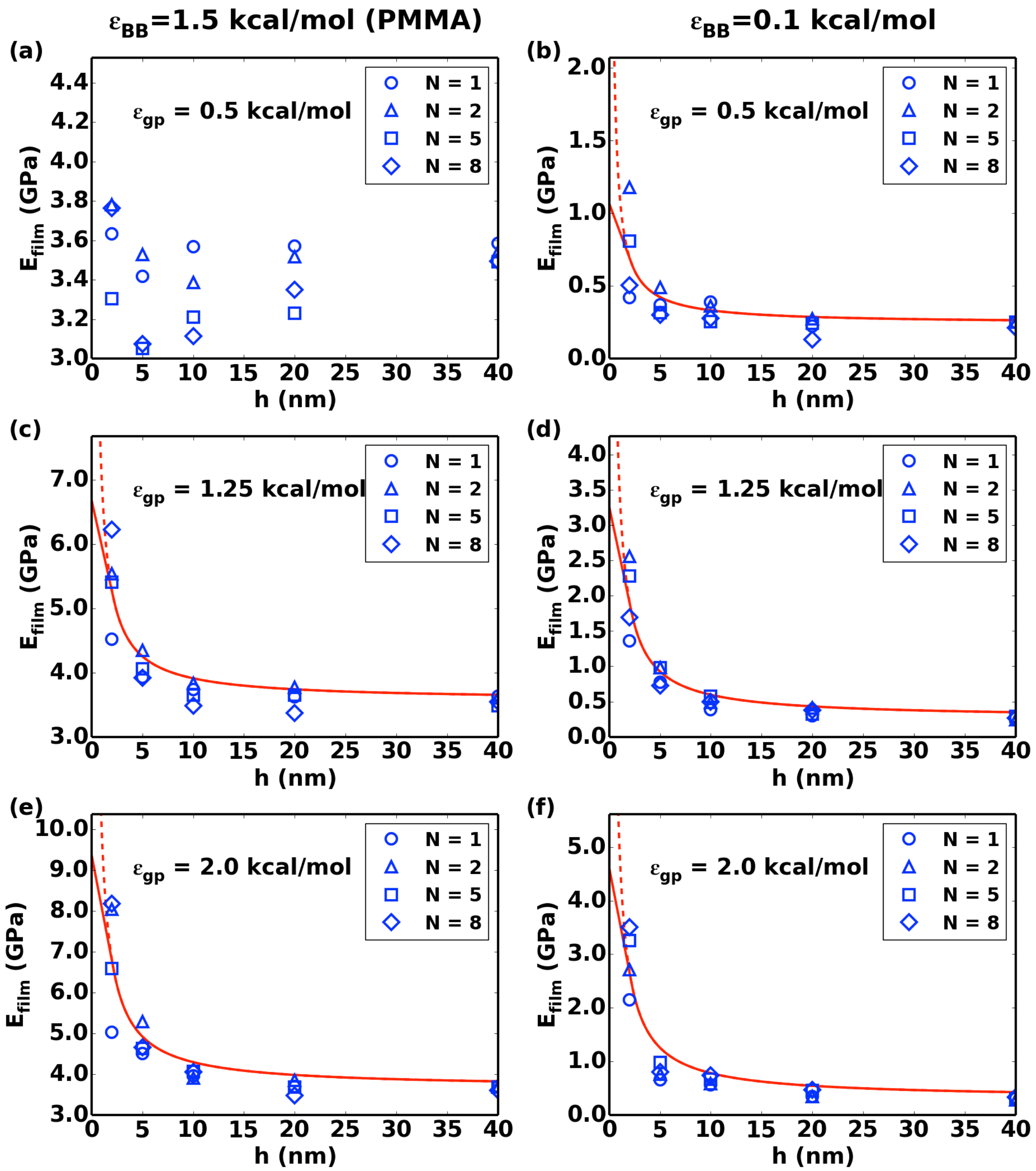

Figure S3. CGMD results and model predictions of the elastic modulus of the confined polymer with ε_{BB} = 1.5 kcal/mol (PMMA) (panel a, c, e) and 0.1 kcal/mol (panel b, d, f) with interfacial interaction strength ε_{gp} = 0.5, 1.25, 2.0 kcal/mol. Solid line is prediction using Equation 11 and dashed line is prediction using Equation 9. *N* is the number of graphene sheets in graphene phase,

h is the thickness of confined polymer phase, E_{film} is the elastic modulus of confined polymer phase. No trend line is provided for panel (a) since the increase in modulus is within the error for this scenario.

Graphene phase elastic modulus

Similar to the method we utilize to calculate stress in polymer phase, virial stress is computed and averaged over all atoms in graphene phase to get stress in graphene layer. Elastic modulus of graphene E_g is then computed from the slope of a linear fit to the stress-strain curve with strain $\varepsilon = 0 - 0.015$. **Figure S4** presents calculated graphene elastic modulus plotted versus number of graphene sheets N. For N>1, the modulus is calculated to be $E_g \sim 300$ GPa, which is in agreement with simulation results on multilayer graphene sheets from our previous study.[4] For systems with N=1, since the graphene flakes in graphene sheets are of finite size, the interfacial shear interaction between graphene and polymer dominates the elastic response of the graphene phase. These are reflected in Figure 3 where reduced modulus is observed with N=1 and E_g increases with increasing ε_{gp}.

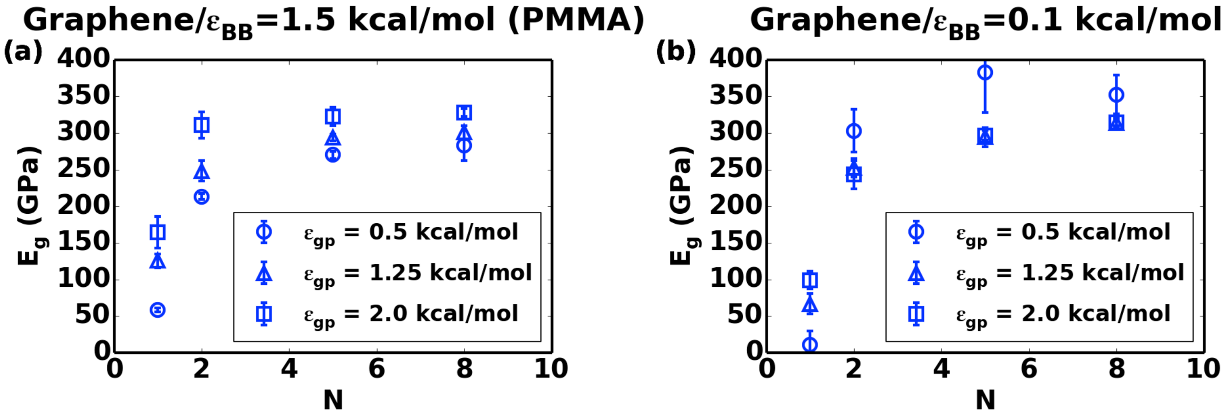

Figure S4. Calculated graphene elastic modulus E_g plotted against number of graphene sheets N for nanocomposites with polymer with ε_{BB} = (a) 1.5 kcal/mol (PMMA) and (b) 0.1 kcal/mol.

1 Soles, C. L. & Ding, Y. Nanoscale polymer processing. *Science* **322**, 689-690 (2008).
2 Jones, R. L., Kumar, S. K., Ho, D. L., Briber, R. M. & Russell, T. P. Chain conformation in ultrathin polymer films. *Nature* **400**, 146-149 (1999).
3 Kumar, S. K., Vacatello, M. & Yoon, D. Y. Off-lattice Monte Carlo simulations of polymer melts confined between two plates. 2. Effects of chain length and plate separation. *Macromolecules* **23**, 2189-2197 (1990).
4 Ruiz, L., Xia, W., Meng, Z. & Keten, S. A coarse-grained model for the mechanical behavior of multi-layer graphene. *Carbon* (2014).
